# Genome sequencing and annotation of two rhizobacteria with antifungal activity: *Pseudomonas tolaasii* strain A46 and *Pseudomonas palleroriana* P61

**DOI:** 10.1128/mra.01385-25

**Published:** 2026-09-14

**Authors:** Freddy Castillo-Alfonso, Marco Polo Carballo-Sánchez, Juan Jose Almaraz-Suarez, Roberto Olivares-Hernández

**Affiliations:** 1 Departamento de Ingenieria Celular y Biocatálisis, Instituto de Biotecnología, Universidad Nacional Autónoma de México, Cuernavaca, Morelos, México; 2 Posgrado en Edafología, Colegio de Postgraduados 61583 , Texcoco, Estado de México, México; 3 Departamento de Procesos y Tecnología, Universidad Autónoma Metropolitana-Cuajimalpa, Ciudad de México, México; DOE Joint Genome Institute, Berkeley, California, USA

**Keywords:** antagonistic rhizobacteria, antifungal activity, secondary metabolite biosynthetic pathways, biological control of soil pathogens

## Abstract

Here, we report the draft genome sequences of *P. tolaasii* A46 and *P. palleroriana* P61, two rhizobacteria previously shown to inhibit *Rhizoctonia solani*. These genomic resources will support future efforts to elucidate the molecular basis of fungal suppression and to assess the biocontrol potential of these *Pseudomonas* strains.

## ANNOUNCEMENT

Soil-borne fungal pathogens, such as *Rhizoctonia solani*, cause severe damage to chili pepper crops and contribute to major yield losses worldwide. Biological control using plant growth-promoting rhizobacteria (PGPR) has emerged as an effective and environmentally friendly alternative to chemical fungicides (1). Among these beneficial microbes, several strains exhibit strong antagonistic activity through the production of antifungal metabolites (2). We report the genome annotation of *P. tolaasii* strain A46 and *P. palleroniana* strain P61, two rhizobacteria previously shown to inhibit *R. solani* in chili seedlings (3).

Genomic DNA was extracted from bacterial cultures grown in nutrient broth for 24 h (28°C, 200 rpm) using the EZ-10 Spin Column Bacterial DNA Mini-Prep Kit (Bio Basic Inc., Canada). A ≈1,500-bp fragment of the 16S rRNA gene was amplified using primers 27F and 1492R . PCR products were purified with the Agencourt AMPure XP Kit and sequenced at MACROGEN (3). The resulting 16S rRNA sequences for each strain were deposited in GenBank (KY933651.1 and KY933652.1).

Libraries were prepared using Covaris DNA fragmentation and size selection. Qualified libraries were converted into DNA nanoballs by rolling-circle replication and sequenced on the DNBSEQ platform (MGI/BGI) using paired-end 150-bp reads. K-mer profiling generated with Jellyfish v1.0.1 (4) revealed a single dominant peak at ≈37× and an estimated genome size of ≈6.52 Mb for both A46 and P61, while the sequencing quality assessment performed with SOAPnuke (RRID:SCR_015025) (5) showed clean, high-quality reads; together with the homogeneous ≈60% guanine–cytosine (GC) patterns and uniform depth observed in the GC–depth plots, these results confirm that both assemblies are of high quality and free of detectable contamination. Clean reads were assembled *de novo* using SOAPdenovo v1.05 (6) and locally optimized using paired-end information. The A46 assembly consisted of 215 scaffolds with a total length of 6,474,022 bp, an N50 of 539,579 bp, and a GC content of 59.67%. The P61 genome comprised 39 scaffolds totaling 6,199,974 bp, with an N50 of 308,887 bp and a GC content of 60.55%. BUSCO v5 (7) analysis using the pseudomonadales_odb10 data set showed high completeness for both genomes, with 97.8% in strain P61 and 98.0% in strain A46, indicating well-assembled and nearly complete genomes. Gene prediction was performed with Glimmer v3.02 (8). Tandem repeats were detected with TRF (9), and functional annotation was carried out using DIAMOND v2.0.7 (10) searches against KEGG (11), COG (12), Uniprot (13), ARDB (14), dbCAN3 (15), and VFDB (16). Genome annotation revealed comparable functional profiles, with A46 encoding 4,878 predicted genes and P61 encoding 4,623. Both strains showed similar proportions of virulence-related genes (8.9% in A46; 9.35% in P61), antibiotic resistance (0.46% and 0.67%, respectively), and CAZy enzymes (3.81% and 4.07%), as well as extensive Swiss-Prot and COG assignments. Overall annotation completeness was high in both genomes, reaching 82.95% for A46 and 86.61% for P61 based on integration of all databases.

AntiSMASH (17) analysis showed that P61 contains multiple NRPS-related biosynthetic clusters, including syringomycin, viscosin, safracin, and fengycin-like pathways, along with siderophore and arylpolyene systems, supporting its strong antifungal potential. Consistent with its broader biosynthetic repertoire, P61 inhibited fungal growth by 49%, whereas A46 showed a lower inhibition of 37% (3).

## Data Availability

The draft genome sequences of *Pseudomonas tolaasii* strain A46 and *Pseudomonas palleroniana* strain P61 have been deposited in GenBank under BioProject accession numbers PRJNA1368354 and PRJNA1368355, respectively. The corresponding whole-genome shotgun (WGS) assemblies are available under accession numbers JCBIYQ000000000 (*P. tolaasii* A46) and JCBIYR000000000 (*P. palleroniana* P61). Raw sequencing reads for both strains have been deposited in the Sequence Read Archive (SRA) under accession numbers SRR36181079 (strain A46) and SRR36181080 (strain P61). Additional supplemental information supporting this study is available at Zenodo under DOI at https://doi.org/10.5281/zenodo.17706700.
